# Substantial increase in the frequency of circulating CD4^+^NKG2D^+^ T cells in patients with cervical intraepithelial neoplasia grade 1

**DOI:** 10.1186/1423-0127-20-60

**Published:** 2013-08-16

**Authors:** Mariel Garcia-Chagollan, Luis F Jave-Suarez, Jesse Haramati, Pedro E Sanchez-Hernandez, Adriana Aguilar-Lemarroy, Miriam R Bueno-Topete, Ana L Pereira-Suarez, Mary Fafutis-Morris, Angel Cid-Arregui, Susana del Toro-Arreola

**Affiliations:** 1 Laboratorio de Inmunología, Departamento de Fisiología, Centro Universitario de Ciencias de la Salud, Universidad de Guadalajara, Sierra Mojada # 950, Colonia Independencia, Guadalajara, Jalisco CP 44340, México; 2 División de Inmunología, Centro de Investigación Biomédica de Occidente, Instituto Mexicano del Seguro Social, Guadalajara, Jalisco, México; 3 Departamento de Biología Molecular y Genómica, Instituto de Enfermedades Crónico-Degenerativas, Centro Universitario de Ciencias de la Salud, Universidad de Guadalajara, Guadalajara, Jalisco, México; 4 Translational Immunology Unit, German Cancer Research Center (DKFZ), Im Neuenheimer Feld 280, Heidelberg 69120, Germany

**Keywords:** NKG2D, CD4^+^ T cells, CIN 1, MICA/B

## Abstract

**Background:**

The NKG2D receptor confers important activating signals to NK cells via ligands expressed during cellular stress and viral infection. This receptor has generated great interest because not only is it expressed on NK cells, but it is also seen in virtually all CD8^+^ cytotoxic T cells and is classically considered absent in CD4^+^ T cells. However, recent studies have identified a distinctive population of CD4^+^ T cells that do express NKG2D, which could represent a particular cytotoxic effector population involved in viral infections and chronic diseases. On the other hand, increased incidence of human papillomavirus-associated lesions in CD4^+^ T cell-immunocompromised individuals suggests that CD4^+^ T cells play a key role in controlling the viral infection. Therefore, this study was focused on identifying the frequency of NKG2D-expressing CD4^+^ T cells in patients with cervical intraepithelial neoplasia (CIN) 1. Additionally, factors influencing CD4^+^NKG2D^+^ T cell expansion were also measured.

**Results:**

Close to 50% of patients with CIN 1 contained at least one of the 37 HPV types detected by our genotyping system. A tendency for increased CD4^+^ T cells and CD8^+^ T cells and decreased NK cells was found in CIN 1 patients. The percentage of circulating CD4^+^ T cells co-expressing the NKG2D receptor significantly increased in women with CIN 1 *versus* control group. Interestingly, the increase of CD4^+^NKG2D^+^ T cells was seen in patients with CIN 1, despite the overall levels of CD4^+^ T cells did not significantly increase. We also found a significant increase of soluble MICB in CIN 1 patients; however, no correlation with the presence of CD4^+^NKG2D^+^ T cells was seen. While TGF-beta was significantly decreased in the group of CIN 1 patients, both TNF-alpha and IL-15 showed a tendency to increase in this group.

**Conclusions:**

Taken together, our results suggest that the significant increase within the CD4^+^NKG2D^+^ T cell population in CIN 1 patients might be the result of a chronic exposure to viral and/or pro-inflammatory factors, and concomitantly might also influence the clearance of CIN 1-type lesion.

## Background

Cervical cancer is one of the most common malignant tumors among women in developing countries [1,2]. Clinical, molecular and epidemiological data have established a strong link between human papillomavirus (HPV) and cervical squamous cell carcinoma development [3-7]. However, before cancer is established, the cervix must go through a series of steps ranging from cervical intraepithelial neoplasia (CIN) grade 1, 2 and 3, before eventually progressing to invasive carcinoma [8-10]. CIN 1, the most benign cervical intraepithelial lesion, will spontaneously regress within 12 to 24 months post-diagnosis of the dysplastic smear in most cases, and this is especially true in those women with non-oncogenic HPV infections [11-15]. It is well accepted that the immune response mediated by CD4^+^ T cells plays an important role in the regression or progression of CIN towards later stages. To make evident the critical involvement of the CD4^+^ T cell response, there is the fact that women infected with human immunodeficiency virus have an increased incidence of squamous intraepithelial lesions, mostly because an increased risk of HPV infection, as well as due to the immunosuppressive state [16-19]. Thus, HPV-specific responses have suggested that CD4^+^ T cells are invaluable effectors used to control of HPV infection, disease regression and clearance [20-22]. Consequently, a number of therapeutic approaches designed to target HPV E6/E7 antigens and the induction of a robust Th1-mediated immune response have been developed for patients diagnosed with either CIN or established cervical cancer [23-26]. Consistent with a protective IFN-γ-producing Th1 phenotype, a shift towards a Th2-type immune response has been associated with cervical cancer progression [27]. Thus, it is clear that Th1-derived cytokines might stimulate an effective cytotoxicity response mediated mainly by CD8^+^ T cells. However, the existence of a unique subset of CD4^+^ T cells endowed with cytolytic capacities has, several decades ago, also been described [28-30], but it has not been until recently that their physiological role has begun to be clarified. Why do some CD4^+^ T cells exhibit cytotoxic behavior? This has been an important question resolved, in part, by the fact that these cells express the activating receptor NKG2D.

NKG2D is a C-type lectin-like immunoreceptor encoded within the NK gene complex on human chromosome 12 [31] with wide expression on NK cells, CD8^+^ T cells and γδ T cells [32,33]. NKG2D is evolutionary conserved in primates and rodents and its symmetric structure consists of a homodimer assembled with transmembrane adaptor molecules, such as DAP10 (in human and mouse) and DAP12 (only in mouse) [34,35]. Upon engagement by natural ligands, such as MHC class I-related chain molecules A and B (MICA and MICB), the complex NKG2D/DAP10 will signal through recruitment of phosphatidyl inositol-3 kinase (PI3K), which will ultimately activate signaling pathways involved in cell survival and release of cytotoxic granules promoting a potent role in tumor destruction or killing of virus-infected cells [36-39]. The cytotoxic signature of NKG2D explains why this receptor had traditionally been confined to NK cells and CD8^+^ cytotoxic T lymphocytes; however, several studies over the last ten years have identified a rare population of CD4^+^ T cells that do express NKG2D, which could represent a particular cytotoxic population involved in viral infections and chronic diseases. For instance, Groh *et al.* reported a substantial number of peripheral and synovial CD4^+^CD28^-^ T cells with expression of NKG2D in patients with rheumatoid arthritis; these CD4^+^NKG2D^+^ T cells apparently influenced by pro-inflammatory cytokines such as IL-15 or TNF-α promoted a cytotoxic response against synoviocytes with anomalous expression of MIC molecules [40]. Therefore, the costimulatory signal triggered by the engagement NKG2D/NKG2D ligands coupled with suboptimal stimulation via TCR will induce important cytokine and cytotoxic responses, thereby self-perpetuating the CD4^+^NKG2D^+^ T cell autoreactivity in rheumatoid arthritis [40,41]. However, the molecular basis influencing the expression of cytotoxicity-related receptors on CD4^+^ T cells remain still under evaluation; however, it is though that chronic antigenic stimulation, such as occurring with some viral infections might lead to NKG2D expression. At least, the existence of a large proportion of CD4^+^NKG2D^+^ T cells has been reported in HTLV-1-associated neurologic disease, as well as in human cytomegalovirus-seropositive individuals [42,43]. Paradoxically, persistent expression of MIC in inflamed tissues in patients with juvenile-onset systemic lupus erythematosus could also promote the expansion of a TGF-β and IL-10-producing CD4^+^NKG2D^+^ T cell population, which would ameliorate the activity of disease [44]. All the above data, apparently contradictory, resulted in the proposal of two distinct populations, one with inflammatory cytokine profile and cytotoxic signature, the other one, a normally-occurring CD4^+^NKG2D^+^ T cell population with immunoregulatory activities [44], although this suppressor population could be exploited by tumors as an strategy to avoid the immune attack. Certainly, substantial number of MIC-dependent CD4^+^NKG2D^+^ T cells has been found in patients with different malignancies, and such population has shown to exert suppressor activities through anti-inflammatory cytokines and Fas ligand-mediated suppression [45]. Thus, it is expected that NKG2D will play a dual role on the particular CD4^+^ T cell population and the decision to adopt a role or the other, will depend in part of the extracellular milieu conditions in which this population is present.

In the particular case of CIN 1, majority of the lesions will spontaneously regress as previously mentioned; however, some of them in which HPV establishes as a chronic infection, will persist and even more, will progress to advanced stages increasing the risk of cervical cancer development. In this scenario, it is firstly important to describe whether the CD4^+^NKG2D^+^ T cell population is also expanded in patients with early cervical neoplasia, and secondly, to explore whether inductor factors of this population are present in the same group of patients. For that reason, here we addressed both goals and we demonstrated that CIN 1 patients show an increase of CD4^+^NKG2D^+^ T cells even when overall levels of CD4^+^ T cells did not increase. We also found a significant increase of soluble MICB in CIN 1 patients; however, no correlation with the presence of CD4^+^NKG2D^+^ T cells was seen. While TGF-β was significantly decreased in the group of CIN 1 patients, both TNF-α and IL-15 showed a tendency to increase (although not significantly) in this group. Therefore, these findings suggest that the expansion of CD4^+^NKG2D^+^ T cells in CIN 1 patients might be under the control of other pro-inflammatory factors still unknown at the moment.

## Methods

### Patients and normal donors

In total, we recruited 33 patients that were first subjected to conventional colposcopic evaluation and finally diagnosed with cervical intraepithelial neoplasia (CIN) grade 1 based on histological analyses. All patients were attended at Gynecology Departments, belonging to Hospital Civil de Guadalajara (OPD) and Centro Médico Nacional de Occidente, IMSS (Guadalajara, México). Thirty age/gender matched healthy volunteers without a history of human papillomavirus (HPV) infection or uterine cervix lesions were also included as control group. Signed informed consent and patient data, including age, and smoking, drug or alcohol use history was given prior to sample collection. The age range of the CIN 1 patients was 20–61 years (mean ± SEM was 34 ± 1.6 years) and that of the control group was 24–61 years (mean ± SEM was 36.5 ± 1.9 years).

### Inclusion and exclusion criteria

Patients were included if they were colposcopically and histopathologically diagnosed as CIN 1. Patients were not included in the study if they had other documented incidences of current inflammatory processes (such as autoimmune or allergic disorders). Control women were negative for HPV infection; they were excluded if they were positive for any HPV genotype.

### Ethical considerations

The study was approved by the local ethical Committee of the Centro Universitario de Ciencias de la Salud, Universidad de Guadalajara (Guadalajara, Mexico), in accordance with the guidelines of the Mexican Official Standard (Norma Oficial Mexicana NOM) and the World Medical Association Declaration of Helsinki (adopted by the 59th WMA General Assembly, Seoul, South Korea, 2008). All women were informed of their rights, the goals of the study and the importance of their participation. The procedures used for collection of samples were identical to those routinely used in the clinical setting and those used for patients not being part of this study.

### Sample collection

Five mL of peripheral blood (PB) was collected into heparin-coated tubes and used for flow cytometry assays. PB was also obtained without heparin in order to separate serum, which was stored at −70°C until analysis by ELISA. Cervical scrape specimens were gently collected from the squamocolumnar junction of the cervix using a sampling brush and were stored at 4°C prior to HPV genotyping.

### HPV genotyping

LINEAR ARRAY® HPV Genotyping Test was performed in accordance with the manufacturer’s recommendations (Roche Diagnostics). Briefly, DNA was extracted from cervical scrapes and amplified in a total volume of 100 μL containing 50 μL of DNA and 50 μL of the master mixture provided by the manufacturer. PCR was performed on a thermocycler T3 (Biometra, Göttingen, Germany) and PCR products were stored at −30°C until use. Colored signals on the strips were read by the naked eye and interpreted according to the LINEAR ARRAY reference guide. Each hybridization strip included two β-globin probe lines (β-globin high and β-globin low), as a positive control for the integrity of the DNA sample.

### Expression of the immunoreceptor NKG2D on CD4^+^ T cell population

CD3 FITC (Santa Cruz Biotech, clone UCH-T1), CD4 PECy5 (Biolegend, clone RPA-T4), CD8 PECy5 (Biolegend, clone RPA-T8), CD56 PECy5 (Biolegend, clone HCD56) and NKG2D PE (Biolegend, clone 1D11) were used to identify the expression of NKG2D on CD4^+^ T cells, CD8^+^ T cells and NK cells in a three-color protocol for flow cytometry. Cells were stained for 45 min at 4°C protected from the light. After incubation, but before washing, erythrocytes in the blood were lysed using BD FACS Lysis Buffer (BD Biosciences). Each tube with 100 μL of blood was diluted with 2 mL of 1× lysis buffer. Tubes were vortexed for ten seconds and then incubated for 15 min protected from the light at room temperature. Cells were then vortexed rapidly and centrifuged at 300 g for 6 min. The lysed erythrocytes and lysis solution were decanted and the pellet was washed with 2 mL of PBS/0.1% sodium azide. Cells were centrifuged again, and resuspended in 500 μL of PBS/0.5% paraformaldehyde. Lymphocytes were distinguished from other mononuclear cells using morphological parameters, namely different coordinates on the forward and side scatter axis, during the flow cytometry scan. Gate was drawn around the lymphocyte population, which was then analyzed separately using the triple marking antibody scheme. A FACSAria flow cytometer (BD Biosciences) was used to acquire ten thousand events in the lymphocyte region for each condition. It is important to mention that this protocol was also extended to calculate the percentages of CD4^+^ T cells, CD8^+^ T cells and NK cells in our two study groups.

### ELISA assays to quantify MICA, MICB, TGF-β, TNF-α, and IL-15

Commercial pre-coated ELISA plates were used to quantify serum MICA, MICB (both from RayBiotech, Inc. Norcross, GA), TGF-β (R&D Systems, Minneapolis, MN), TNF-α, and IL-15 (both from BioLegend, San Diego, CA). Immediately prior to use, sera were thawed from −70°C storage. All samples were run by duplicate and values reported as average of the duplicates. The manufacturer’s instructions were followed for the assays. Briefly, plates were washed and samples and standards were pipetted into the wells. Plates were sealed and incubated overnight at 4°C. Biotinylated detection antibodies were added and incubated for 1 hour at room temperature on an orbital shaker. Avidin-HRP detection complexes were added and incubated. Next, the plates were incubated with the chromogenic substrate solution and covered from the light. Stop solution was transferred to each well and samples were read within 30 minutes. Absorbance was calculated as absorbance at 450 nm minus absorbance at 570 nm. The values of blanks were subtracted from sample values and curve fitting was calculated using a 4-parameter logistic curve-fitting algorithm (CurveExpert Basic, v 1.4).

### Statistical analysis

Results of soluble MICA, MICB, TGF-β, TNF-α, and IL-15, as well as percentages of NKG2D expression on T cells and NK cells were expressed as mean ± SEM. Data were tested for normal distribution using Shapiro-Wilk test for small sample size. Due to the non-normal distribution, data were analyzed with Mann–Whitney U test. All the statistical analyses were performed considering *p* < 0.05 to be significant using SPSS 15.0 software (SPSS, Chicago, IL, USA).

## Results

### Distribution of human papillomavirus (HPV) genotypes

Some studies have reported a HPV prevalence ranged from 67.1% to 68.3% for Europe, South/Central America [46]. Using a commercial kit for detection of 37 high- and low-risk HPV genotypes we found that close to 50% of the patients with cervical intraepithelial neoplasia (CIN) 1 contained at least one of the 37 HPV genotypes. With regard to the HPV genotypes of infected patients, we found a wide dispersion of different strains of HPV. Infection with high-risk HPV was present in 75% of CIN 1 patients that were positive for at least one viral strain (Table 1). Although the most common strains of high-risk and low-risk HPV are 16, 18 and 6, 11, respectively [46,47], in our viral typing the most common strain of high-risk HPV was 51, and the most common low-risk strain was 84 (Table 1). Interestingly, 37.5% of CIN 1 patients infected with HPV presented more than one strain of HPV (co-infection cases). In these cases, it is interesting to note that 100% of the patients were co-infected with at least one low-risk viral sub-type.

**Table 1:** HPV genotypes in patients with histologically confirmed diagnosis of CIN 1

**Patient with diagnosis of CIN-1**	**Age**	**Infection**	**HPV infection (Viral subtyping)**	
			**Low risk**	**High risk**
1	37	Negative		
2	30	Negative		
3	25			51
4	38			51
5	20			16
6	44			58
7	25	Negative		
8	34			59
9	49	Negative		
10	61	Negative		
11	24	Negative		
12	37	Negative		
13	23		84, 72, 62, 61	
14	42		62	
15	36		6, 53, 61, 84 CP6108	
16	41			51
17	29		CP6018	
18	48			39
19	23	Negative		
20	23		84	16
21	34	Negative		
22	28			33
23	38		42	16
24	43	Negative		
25	47	Negative		
26	29		66	51
27	37	Negative		
28	28	Negative		
29	23	Negative		
30	27		84	35
31	25	Negative		
32	30	Negative		
33	44	Negative		

### Changes in numbers of lymphocyte populations

**Figure 1:**
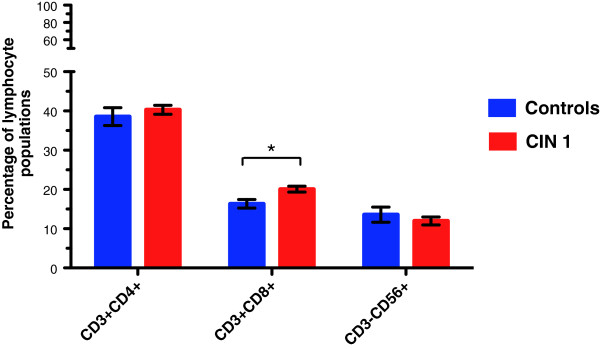
**CD8**^**+ **^**T cells are significantly increased in CIN 1 patients.** Flow cytometric analysis of peripheral blood in CIN 1 patients or normal donors was performed in order to evaluate variations in the percentages of T cells and NK cells. Gate was drawn around the lymphocyte population, which was then analyzed separately with the corresponding antibodies (CD3^+^ FITC/CD4^+^ PECy5, CD3^+^ FITC/CD8^+^ PECy5, and CD3^-^ FITC/CD56^+^ PECy5 for detection of CD4 T cells, CD8 T cells and NK cells, respectively). Analysis from the lymphocyte gate shows that both CD4^+^ T cells and CD8^+^ T cells were increased in CIN 1 patients, while NK cells were diminished; however, we only observed a significant increase in the CD8^+^ T cell population. Statistical analysis was performed through Mann–Whitney U test and data were expressed as mean ± SEM; *p = 0.015.

An effective T cell response might be important for CIN 1 regression. Although, the frequency of the different lymphocyte populations in women with CIN 1 has been well documented in the literature, in this study we wanted to re-evaluate the frequencies of CD4^+^ T cells, CD8^+^ T cells, and NK cells, in order to rule out whether a possible increase in the pool of NKG2D-expressing CD4^+^ T cells might be due to variations in the frequency of these populations. Patients with CIN 1 lesions (n = 33) and controls (n = 30) were examined. Lymphocyte counts (expressed as percentages) were performed using the cytograms, according to the characteristics of forward scatter (FS) and side scatter (SS). Thus, in CIN 1 patients, we found a mean of 27.07 ± 1.22% (range 15.8 ± 45.4%), whereas in healthy controls a mean of 28.61 ± 1.35% (range 23.2 ± 34.2%) was found. We observed a tendency for increased CD4^+^ T cells and CD8^+^ T cells and decreased NK cells in CIN 1 patients. CD4^+^ T cells were present at 38% of the lymphocyte gate of peripheral blood mononuclear cells (PBMCs) in healthy controls and 40% in CIN 1 patients. CD8^+^ T cells increased from 16% to 20% in patients *versus* controls. CD56^+^ (NK) cells decreased from 13% to 11%. Only the increase in CD8^+^ T cells was significant (*p* = 0.015) as can be seen in Figure 1.

### Increased CD4^+^NKG2D^+^ T cells and soluble MICB in CIN 1 patients

**Figure 2:**
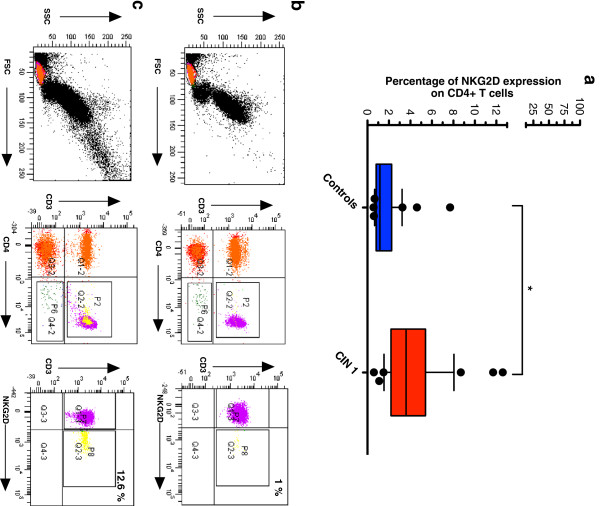
**NKG2D-expressing CD4**^**+ **^**T cells significantly increase in patients with CIN 1.** Three-color flow cytometric analysis to detect the expression of NKG2D on peripheral CD4 T cells was carried out in CIN 1 patients and control donors. Cells from the lymphocyte gate were subgated based on CD3 expression and CD4 co-expression. Then, changes in NKG2D expression on CD4^+^ T cell populations were evaluated. The given percentages reflect the portion of cells positive for NKG2D within the given sub-population. There was a significant increase in CD4^+^NKG2D^+^ T cells in the group of CIN 1 patients when compared with normal donors **(a)**. Representative experiment is showed in **(b)**, CD3^+^CD4^+^ cells were divided into two populations: NKG2D^-^ and NKG2D^+^ (gate P7 and P8, respectively). It can be clearly seen a control donor practically negative for CD4^+^NKG2D^+^ T cells. The other example in **(c)** shows a CIN 1 patient with a high percentage of CD4^+^NKG2D^+^ T cells. Statistical analysis was performed through Mann–Whitney U test and data are expressed as median, which are represented as horizontal lines and 10th and 90th percentiles as whiskers. Extreme values are also showed (•); *p < 0.001.

**Figure 3:**
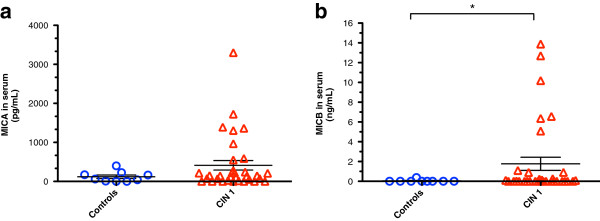
**The highest levels of soluble MICA and MICB are seen in CIN 1 patients.** Levels of soluble MICA and MICB were detected using commercial ELISA kits. The highest levels for both MICA **(a)** and MICB **(b)** were found in CIN 1 patients when compared to healthy controls. While the group of CIN 1 patients showed only a non-significant trend to high soluble MICA, the levels of soluble MICB were significantly augmented in this group *versus* controls. Statistical analysis was performed through one-tailed Mann–Whitney U test and data are expressed as mean ± SEM (horizontal lines). Absorbance values are shown as pg/mL (MICA) and ng/mL (MICB); *p = 0.03.

CD4^+^ T cells that express NKG2D may have either cytotoxic or immunoregulatory roles. In this study, CD4^+^NKG2D^+^ T cells were found to be significantly increased in CIN 1 patients *versus* controls (Figure 2a). Cells from the lymphocyte gate were gated according to CD3 and CD4 co-expression and NKG2D was measured on the double positive CD3^+^CD4^+^ cells in controls (Figure 2b) and patients (Figure 2c). CD4^+^NKG2D^+^ T cells in CIN 1 patients were significantly increased *versus* controls (3.60% and 1.16%, respectively; *p <* 0.001). Interestingly, absolute NKG2D levels were found to decrease in CD8^+^ T cells and NK cells from CIN 1 samples (data not shown). In accordance with the fact that binding of soluble MIC molecules can lead to NKG2D down-regulation, soluble MICB was found to be significantly increased in CIN 1 patients (1.8 ng/mL *versus* 0.04 ng/mL; *p* = 0.03) as shown in Figure 3b. While soluble MICA levels visually increased, this result was not significant (413 pg/mL *versus* 119 pg/mL; *p* = 0.18) as shown in Figure 3a.

### Soluble levels of TGF-β, TNF-α, and IL-15

**Figure 4:**
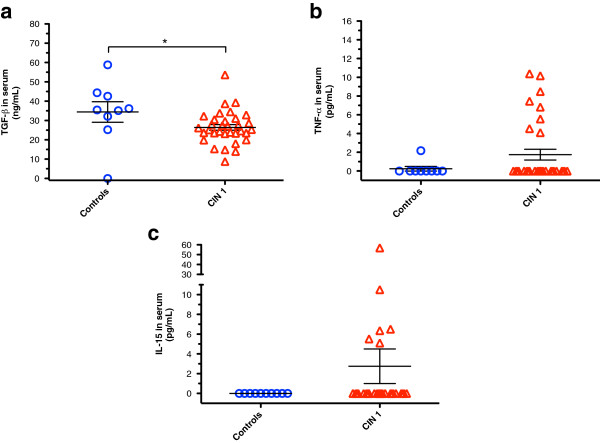
**Trend to decrease TGF-β and increase pro-inflammatory cytokines is seen in CIN 1 patients.** Serum profiles of TGF- β, TNF-α, and IL-15 in CIN 1 patients and control donors were quantified using commercial ELISA kits. While serum levels of TGF-β **(a)** were significantly lower in CIN 1 patients *versus* control group, TNF-α **(b)** and IL-15 **(c)** showed a trend to be increased, although we did not find statistical differences. Statistical analysis was performed through Mann–Whitney U test and data are expressed as mean ± SEM (horizontal lines). Absorbance values are shown as pg/mL (TNF-α and IL-15) and ng/mL (TGF-β); *p = 0.014.

With CD4^+^NKG2D^+^ T cell population being increased in CIN 1 patients, it would appear that a less quiescent cellular milieu was present, as it is known that exposure to pro-inflammatory cytokines can induce the expression of NKG2D on CD4^+^ T cells [40]. In accordance with this, anti-inflammatory TGF-β was found to decrease from 35 pg/mL (in controls) to 26 pg/mL in CIN 1 patients; *p* = 0.014 (Figure 4a). In turn, non-significant increases in pro-inflammatory TNF-α (from 0.2 pg/mL to 1.8 pg/mL; *p* = 0.31) and IL-15 (undetectable in controls) were found (Figure 4b and 4c, respectively). Interestingly, the variation within the control group was very low for TNF-α and IL-15, while CIN 1 patients displayed extreme deviation. The majority of CIN 1 patients had TNF-α and IL-15 levels similar to those of the controls; however, a minority of patients presented levels sharply higher. This might reflect a more chronic local inflammatory state that presupposes a minority of CIN 1 patients towards the development of chronic infections and eventually to cervical cancer. It is notable that 3/6 patients who displayed the highest IL-15 levels, also ranked as the highest producers of TNF-α.

## Discussion

The infection with genital human papillomavirus (HPV) is very common in sexually active women; however most HPV infections are temporary, clearing within 12 to 24 months after diagnosis of the dysplastic smear or, in fact, in a shorter period of time in those women with non-oncogenic HPV infections [11,14,15]. Clinical manifestations of low-risk HPV infections (*i.e.* HPV6 and HPV11) are commonly genital warts [48]; infections with high-risk HPV types (*i.e.* HPV16 and HPV18) are associated with the occurrence of cervical intraepithelial neoplasia (CIN). CIN 1, the grade of the lesion in the women in this study, is the most benign lesion and rarely develops into cervical cancer [49,50]. While the main subject of this study was focused on CD4^+^NKG2D^+^ T cells and potential factors associated with their expansion in patients with CIN 1, our first step was to confirm HPV infection and to identify the HPV genotypes present in our group of patients. We found that close to 50% of the patients were positive for at least one of the 37 HPV types detected by our genotyping system. Initially, this result was cause for concern and led us to question the relatively low percentage of HPV infection, as, based on gynecological examination, a higher number of HPV-infected patients would have been expected. Although there are many potential explanations for this result, one likely explanation is the possibility that a higher number of patients with CIN 1 than recorded by the DNA-based test were actually positive for any of the HPV genotypes. This is based mainly on the fact that some of the patients reported as negative via the DNA-based test for viral infection presented persistent lesions up to 12 months after initial examination (data not shown). Another reason to support the diagnosis of a HPV infection (in the absence of a positive test score) was based on colposcopic features suggestive of HPV infection. Therefore, a conceivable explanation for the low frequency of HPV might be largely attributed to the accuracy of our viral typing method (*i.e.* unable to detect all samples with low viral load) or another possibility might be the infection with other strains of HPV, different than those detected by our system.

An interesting result has been the finding that low risk viral types are increased in CIN 1 patients with multiple viral infections. If we look at our patients infected with only one viral strain (Table 1), we see that high-risk strains predominate at 4:1 ratio (80% of the CIN 1 patients infected with a single strain are positive for high-risk strains). This is in line with previous research that has shown that in women negative for HPV, the cumulative probability of acquiring an oncogenic HPV strain during a 12-month follow-up period was 0.32, compared with 0.18 for non-oncogenic strains [51]. This argues that either high-risk strains have a higher prevalence in the general population, or that high-risk strains have a greater “staying power” in patients. However, whatever the case, the vast majority of HPV-infected women mount an effective immune response to assist the infection clearance without medical intervention [12].

It is well known that CD4^+^ T cell-regulated cellular immune response plays a central role in the control and resolution of HPV-associated cervical lesions [20-22]. For instance, CD4^+^ T cells polarized into a Th1 phenotype secrete consistent amounts of IFN-γ, creating a milieu in which CD8^+^ T cells are activated to generate an effective cytotoxic response against infected cells. This concept is supported by the fact that a shift from Th1- towards a Th2-type immune response is associated with cervical cancer progression [27]. Although HPV life cycle does not seem to cause apparent systemic viraemia and the control of the viral infection is practically confined to the local immune response, several studies have shown a co-dependency between local T cell-mediated responses and those mediated by peripheral blood T cells [52]. The role of systemic immunity is exemplified in women spontaneously resolving CIN 1; in these patients it has been shown that T cells proliferating in response to HPV16 E2 secrete high levels of IFN-γ, which is consistent with a Th1 response [53]. In the present study, we did not perform assays to test the functionality of the peripheral T cell pool; our goal was merely to re-evaluate the frequencies of CD4^+^ T cells, CD8^+^ T cells, and NK cells in order to rule out whether a possible increase in the pool of NKG2D-expressing CD4^+^ T cells might be due to variations in the frequency of the above-mentioned populations. However, the fact that the potent regulatory cytokine TGF-β was significantly decreased in CIN 1 patients *versus* the control group might suggest the predominance of a less quiescent cellular milieu, which in turn might facilitate the generation and expansion of a systemic CD8^+^ T cell-mediated response. An example of the immunoregulatory role of TGF-β during the development of CD8^+^ T cell responses is clearly depicted in a murine model with intracellular bacterial infection. In this study, Sanjabi *et al.* show that the number of effector CD8^+^ T cells following *Listeria* infection is under strict control of TGF-β and IL-15, which exerted contrasting effects during clonal expansion and contraction phases. While TGF-β supported apoptosis of effector CD8^+^ T cells, IL-15 maintained survival of CD8^+^ T cells during the contraction phase [54]. As mentioned above, in our present study we observed on one hand, that the level of TGF-β was significantly reduced in CIN 1 patients (consistent with the significant increase of systemic CD8^+^ T cells); while on the other hand, we also found an increase of IL-15 in this group, although this result was not significant. However, our measurement system was limited to soluble IL-15 in serum; thus, we cannot strictly exclude the possibility that membrane-bound IL-15 on monocytes, for instance, might influence the survival of virus-specific CD8^+^ T cells through a complex mechanism termed trans-presentation, which tightly regulates IL-15 responses in NK cells and CD8^+^ T cells [55-57]. Likewise, it will be important to understand further the role of IL-15 in the progression of CIN advancing to cervical cancer, as this cytokine has also, paradoxically, been implicated in cervical cancer immune escape through its role in the over-expression of the inhibitory receptor CD94/NKG2A on tumor-infiltrating CD8^+^ T lymphocytes [58].

Despite the fact that the role of IL-15 is well established on cytotoxic lymphocytes (namely CD8^+^ T cells and NK cells), there is also evidence that an atypical subset of CD4^+^ T cells endowed with apparent cytotoxic capabilities strictly depend on IL-15 for their expansion. This has been documented in patients with granulomatosis with polyangiitis (a rare disease characterized by vasculitis), in which IL-15 trans-presented by monocytes/macrophages contributes to the survival and proliferation of NKG2D-expressing CD4^+^ T cells, which are the main mediators of the resulting endothelial cell damage [59]. The innate NK-activating receptor had traditionally been restricted to NK cells, CD8^+^ T cells, and γδ^+^ T cells [32,33]; however, in recent years there has been a wealth of evidence that strongly supports the existence of this receptor on CD4^+^ T cells, conferring an important innate-like cytotoxic signature to this particular population. CD4^+^NKG2D^+^ T cells have been seen in chronic viral infections [42,43], as well as in autoimmune disorders, in which these cells may intensify the clinical manifestations after TCR and NKG2D engagement [40,41,60,61]. Contradictory evidence has also tagged this particular population with an immunosuppressive role in patients with cancer [46]. Thus, these apparently confounding data have led to the proposal that two different CD4^+^NKG2D^+^ T cell subsets might be operating in different clinical settings [45]. In our present work, the substantial increase of CD4^+^NKG2D^+^ T cells (particularly observed in CIN 1 patients) is noteworthy. The biological significance of these cells in our CIN 1 patients is unknown at the moment, as we do not yet have any functional evidence to tag this cell population with either pro-inflammatory or regulatory properties. However, it is important to remark that a high percentage of the patients included in our study (almost 80%) were followed up for at least 12 months and almost all the patients cleared their lesions without progressing to more advanced stages (data not shown). Moreover, the few patients that had shown long-term persistence of their lesions were clinically confirmed, at the end of the follow-up period, to be free from disease. Particularly notable is the fact that one of these patients, who also had a bacterial infection during the 4th follow-up ambulatory visit, showed the highest percentage of CD4^+^NKG2D^+^ T cells (12.6%). Despite the fact that we only measured this particular T cell population at the beginning of our study, it is feasible to speculate that persistent HPV lesions might create a privileged setting in which the expression of NKG2D on CD4^+^ T cells would be favored by an imbalance between anti- and pro-inflammatory stimuli. Interestingly, Lee *et al.* have provided evidence showing that TGF-β1 in plasma taken from patients with lung cancer or colorectal cancer impairs NK cell activity via NKG2D down-regulation [62]. Thus, the significant decrease of TGF-β might facilitate, in part, the increase of CD4^+^NKG2D^+^ T cells observed in our CIN 1 patients. Additionally, it has also been demonstrated that soluble MHC class I-related chain molecule A (MICA) is associated with NKG2D down-regulation on NK cells and CD8^+^ T cells is several malignant tumors [63-65]. The biological impact that soluble MICA and MICB might exert on the particular subset of CD4^+^NKG2D^+^ T cells is not well understood, but it has been proposed that pro-inflammatory cytokines may neutralize the NKG2D down-regulation induced by soluble MIC molecules in patients with autoimmune disease [40]. Thus, it is noteworthy that our CIN 1 patients had notably higher amounts of soluble MICA and MICB than the range seen in the control group (although only MICB yielded a significant difference); however, we did not observe any resulting NKG2D down-regulation on CD4^+^ T cells, supporting the idea that the observed pro-inflammatory milieu might be contributing to the maintenance of CD4^+^NKG2D^+^ T cells during the regression of CIN 1.

Finally, the question remains as to why, in only some cases, HPV persists as a chronic infection and may advance from CIN 1 to CIN 2/3 and eventually to cervical cancer. Given our results, it is clear that more work is still needed to elucidate whether pro-inflammatory or regulatory activities are the main feature of CD4^+^NKG2D^+^ T cells in the natural history of cervical intraepithelial neoplasia grade 1.

## Conclusions

Taken together, our results suggest that the significant increase within the CD4^+^NKG2D^+^ T cell population in CIN 1 patients might be the result of a chronic exposure to viral and/or pro-inflammatory factors, and concomitantly might also influence the clearance of CIN 1-type lesion.

## Abbreviations

HPV: Human Papilloma Virus; MICA: MHC class I-related chain A; MICB: MHC class I-related chain B; NKG2D: Natural-killer group 2, member D; CIN: Cervical intraepithelial neoplasia.

## Competing interests

The authors declare that they have no competing interests.

## Authors’ contributions

MGC performed all he experimental work described in the study, searched scientific literature, and contributed with figures. JH and PESH helped with ELISA assays and assisted the statistical analyses. LFJS and AAL contributed with flow cytometry experiments and HPV genotyping. MRBT, ALPS, and MFM contributed with scientific ideas and research. ACA participated in the design of the study and contributed to the review of the manuscript. STA conceived and designed the theoretical framework of the study, provided scientific guidance throughout the project and wrote the manuscript. All authors read and approved the final manuscript.
